# TransMA: an explainable multi-modal deep learning model for predicting properties of ionizable lipid nanoparticles in mRNA delivery

**DOI:** 10.1093/bib/bbaf307

**Published:** 2025-06-29

**Authors:** Kun Wu, Zixu Wang, Xiulong Yang, Yangyang Chen, Fulvio Mastrogiovanni, Jialu Zhang, Lizhuang Liu

**Affiliations:** Shanghai Advanced Research Institute, Chinese Academy of Sciences, 99 Haike Road, Shanghai 201210, China; University of Chinese Academy of Sciences, No. 1 Yanqihu East Rd, Huairou District, Beijing 101408, China; Department of Computer Science, University of Tsukuba, 1-1-1 Tennodai, Tsukuba, Ibaraki 305-8577, Japan; Hubei Provincial Key Laboratory of Artificial Intelligence and Smart Learning, Central China Normal University, 152 Luoyu Road, Hongshan District, Wuhan 430079, Hubei Province, China; School of Computer Science, Central China Normal University, 152 Luoyu Road, Hongshan District, Wuhan 430079, Hubei Province, China; Department of Computer Science, University of Tsukuba, 1-1-1 Tennodai, Tsukuba, Ibaraki 305-8577, Japan; Robotics, Brain, and Cognitive Sciences Unit, Istituto Italiano di Tecnologia, Via Morego 30, 16163 Genoa, Italy; Department of Informatics, Bioengineering, Robotics and Systems Engineering, University of Genoa, Viaall'Opera Pia 11A, 16145 Genoa, Italy; Shanghai Advanced Research Institute, Chinese Academy of Sciences, 99 Haike Road, Shanghai 201210, China; Shanghai Advanced Research Institute, Chinese Academy of Sciences, 99 Haike Road, Shanghai 201210, China

**Keywords:** ionizable lipid nanoparticles, multimodal molecular structure, interpretability, transfection cliffs

## Abstract

As the primary messenger RNA (mRNA) delivery vehicles, ionizable lipid nanoparticles (LNPs) exhibit excellent safety, high transfection efficiency, and strong immune response induction. However, the screening process for LNPs is time-consuming and costly. To expedite the identification of high-transfection-efficiency mRNA drug delivery systems, we propose an explainable LNPs transfection efficiency prediction model, called TransMA. TransMA employs a multimodal molecular structure fusion architecture, wherein the fine-grained atomic spatial relationship extractor named molecule 3D Transformer captures three-dimensional spatial features of the molecule, and the coarse-grained atomic sequence extractor named molecule Mamba captures one-dimensional molecular features. We design the mol-attention mechanism block, enabling it to align coarse and fine-grained atomic features and capture relationships between atomic spatial and sequential structures. TransMA achieves state-of-the-art performance in predicting transfection efficiency using the scaffold and cliff data splitting methods on the current largest LNPs dataset, including Hela and RAW cell lines. Moreover, we find that TransMA captures the relationship between subtle structural changes and significant transfection efficiency variations, providing valuable insights for LNPs design. Additionally, TransMA’s predictions on external transfection efficiency data maintain a consistent order with actual transfection efficiencies, demonstrating its robust generalization capability. We hope that high-accuracy transfection prediction models in the future can aid in LNPs design and initial screening, thereby assisting in accelerating the mRNA design process.

## Introduction

Messenger RNA (mRNA)-based technologies hold promise for therapeutic applications in fields such as viral vaccines, protein replacement therapies, cancer immunotherapies, and genome editing [1–6], and have the potential to reshape the landscape of life science research and medicine [7–9]. However, achieving targeted delivery and intracellular release from endosomes remains challenging for mRNA delivery systems, emphasizing the critical demand for safe and effective mRNA delivery materials. Specifically, lipid nanoparticles (LNPs) have undergone extensive investigation and have successfully transitioned into clinical use for the delivery of mRNA [10–12]. For example, mRNA1273 and BNT162b21 utilize LNPs for the delivery of antigen mRNA [13–15]. LNPs consist of four components: ionizable lipid, phospholipid, cholesterol, and PEGylated lipid. Among these, the ionizable lipid component holds the highest molar ratio, which determines the formulation’s delivery efficiency and stability, serving as the core structure of LNPs [16]. Therefore, the key to selecting for efficient LNPs lies in the selection of the ionizable lipid [17, 18].

Although component chemistry methods based on three-component reactions (3-CR) [19, 20] and four-component reactions (4-CR) [21] can synthesize a number of ionizable lipids, manually testing the transfection efficiency of each synthesized lipid is time-consuming and costly [22, 23]. Several works have shown that artificial intelligence represented by machine learning and deep learning can achieve automatic prediction of LNPs transfection efficiency. For instance, Ding *et al*. [9] employed four machine learning methods—support vector machine, random forest, eXtreme gradient boosting, and multilayer perceptron—to classify the transfection efficiency of 572 LNPs into two categories, achieving a classification accuracy of 98$\%$. AGILE [24] is a prediction platform for LNPs transfection efficiency based on graph convolutional neural networks (CNNs) and pretraining, achieving a mean squared error (MSE) of $\sim $6 in predicting LNPs transfection efficiency. TransLNP [25], which is a model based on Transformer architecture and data balancing, achieved an MSE of $\sim $5 on the AGILE dataset.

Despite significant progress in previous works, it must be acknowledged that there are notable limitations in achieving automatic prediction of LNPs transfection efficiency based on machine learning and deep learning. These limitations hinder the accuracy and generalizability of the models. These limitations include (i) **The lack of multimodal information interaction.** Previous works only extract single-modal information from molecules, which limits their ability to capture Quantitative Structure-Activity Relationships between ionizable lipids and transfection efficiency. For instance, chemberta [26] relies on one-dimensional representations to predict molecular properties. Integrating multimodal information from molecules is crucial for further improving prediction accuracy. (ii) **Previous works lacks interpretability.** Although previous works can predict transfection efficiency accurately, it is unclear which atoms play a key role in this process. Explainable AI (xAI) is key for molecular property prediction, supporting drug screening and discovery [27]. While *post hoc* methods like SHAP [28] and LIME [29] work across models, they struggle with complex ones like Transformer and Mamba fusion models. Transformers’ multi-head attention makes it hard to capture layer details, and Mamba fusion models’ integration of different architectures adds complexity, making decision interpretation challenging [30]. These methods also face computational and transparency issues. A *pre hoc* explanation approach, integrated from model construction, is crucial for improving interpretability in such complex models. (iii) **Limited attention to the impact of transfection cliffs.** Pairs of molecules that are structurally very similar but have significantly different transfection efficiencies—known as transfection cliffs—capture the knowledge hidden in the elusive structure–property relationships but have received limited attention.

To address the challenges, we introduce an explainable LNPs transfection efficiency prediction model called TransMA, which adopts a multimodal molecular structure fusion architecture. TransMA integrates 3D geometric information and 1D atomic sequence information of molecules to predict LNPs transfection efficiency. A self-attention mechanism SE(3) Transformer architecture named molecule 3D Transformer is employed to extract 3D geometric information. Molecule 3D Transformer is pretrained by reconstructing atomic 3D coordinates and masked atom prediction. To extract atomic sequence information, the state space model designated as molecule Mamba is presented to obtain coarse-grained atomic sequence information. By constructing multilevel molecular structure data pairs, the model jointly learns the multidimensional structural features of molecules. Specifically, we design the mol-attention mechanism block to align and concatenate the fusion features. The fusion features include atomic sequence distribution information, atomic coordinates information, relative atomic positions information, and types of bonds between atoms information. Compared with state-of-the-art molecular graph convolutional networks and Transformer models, TransMA demonstrates the best performance, reducing MSE by 43$\%$ compared with the baseline model AGILE and by 35$\%$ compared with TransLNP. Additionally, TransMA possesses interpretability to better understand the mapping relationship between molecular structure and biochemical properties [31, 32]. The mol-attention mechanism block not only integrates multimodal molecular features but also reveal critical sites that influence transfection efficiency. Transfection cliffs represent pairs of molecules that are structurally similar but exhibit substantial differences in transfection efficiency. Extracting these transfection cliff data can help us understand the elusive transfection efficiency. A total of 4267 and 2104 transfection cliffs are screened from Hela and RAW 264.7 cell lines, respectively, constituting 68$\%$ and 81$\%$ of all data, indicating the widespread occurrence of transfection cliffs. Our findings is that atoms with high attention scores calculated by the mol-attention mechanism block corresponded to key atoms in transfection cliff pairs. This finding indicates that significant differences in transfection efficiency caused by subtle structural changes. To test the generalization capability of TransMA, the results demonstrate that TransMA’s predicted values maintain a consistent ranking with the actual transfection efficiency values without training on the external dataset.

In this study, our primary contributions can be summarized as follows:

1. We propose an explainable LNPs transfection efficiency prediction model, named TransMA, which adopts a multimodal molecular structure fusion architecture. The fine-grained atomic spatial relationship extractor named molecule 3D Transformer captures molecular 3D geometric features, while the coarse-grained atomic sequence extractor named molecule Mamba captures 1D SMILES representation molecular features.
2. TransMA achieves state-of-the-art performance on the current largest LNPs dataset, showing significant improvement across various metrics compared with previous methods.
3. TransMA demonstrates strong interpretability. We construct 4267 and 2104 pairs of transfection cliffs in Hela and RAW 264.7 cell lines to validate the model’s identification of key molecular structures. While validating TransMA’s prediction results, we find that it can detect substantial transfection efficiency differences caused by minor structural variations.
4. We collect independent external test data from several sources. TransMA’s predicted values consistently maintain the same order as the actual transfection efficiencies, highlighting its robust generalization capacity.

## Materials and methods

### The method of TransMA to predict LNPs transfection efficiency

**Figure 1:**
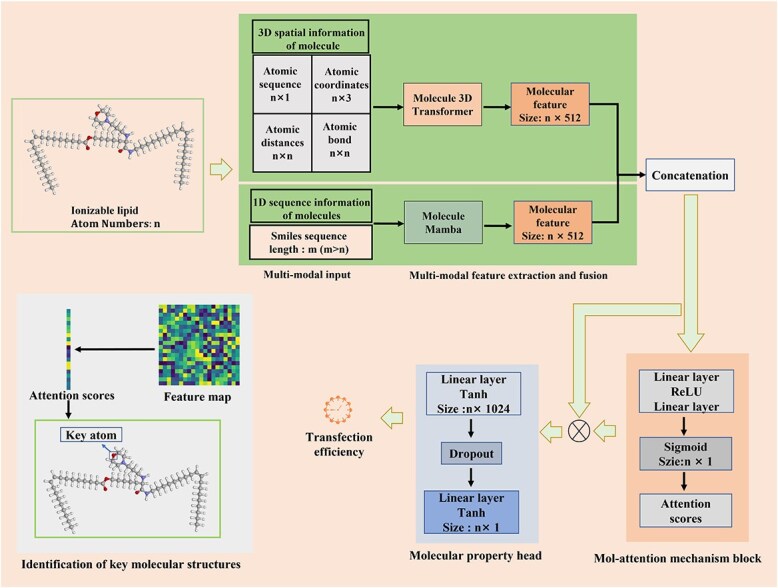
The overall architecture of TransMA processes multimodal structural information of ionizable lipids, including 3D molecular features and 1D SMILES representations, using a Molecule 3D Transformer and Molecule Mamba module, and integrates the extracted spatial and sequential features through a mol-attention mechanism to predict transfection efficiency.

Deep learning is a powerful tool for accelerating research in the molecular field [33, 34]. We develop an explainable deep learning approach to predict the transfection efficiency of LNPs. This model not only achieves high-precision predictions but also elucidates the key molecular structures that the model focuses on. The overall architecture of TransMA is illustrated in Fig. 1. The TransMA framework consists of three components: molecule 3D Transformer, molecule Mamba, and mol-attention mechanism block.

#### Molecule 3D transformer

**Figure 2:**
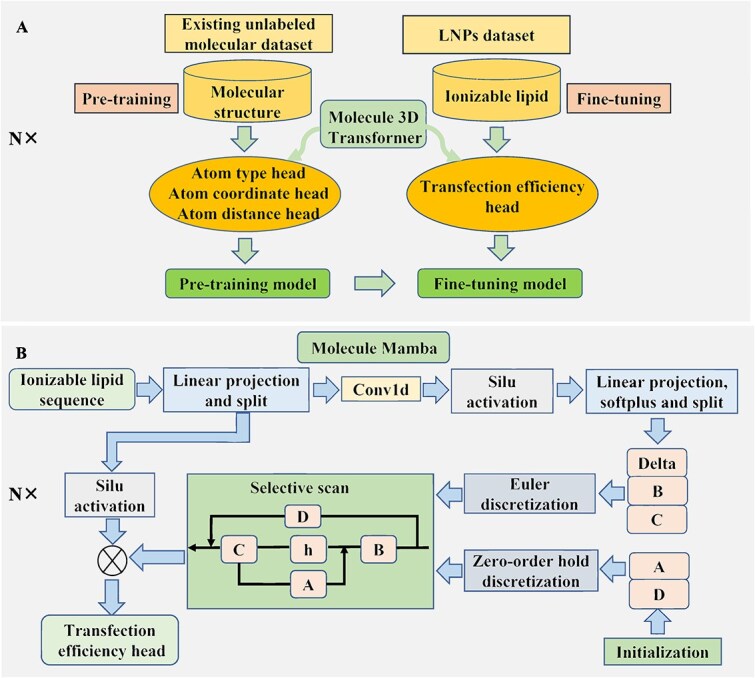
The overall architectures of the Molecule 3D Transformer (subfigure A) and Molecule Mamba (subfigure B) involve pretraining tasks such as masked language modeling and noisy coordinate prediction for the Transformer, and a selective scan operation with a discretized SSM equation for molecule mamba, which extracts high-dimensional features from SMILES representations to predict transfection efficiency.

The molecule 3D Transformer is employed to extract the three-dimensional structural information features of molecules. These three-dimensional structural details include the atomic type sequences, atomic three-dimensional coordinates, Euclidean distance matrices between atoms, and matrices representing the types of bonds between atoms in ionizable lipid molecules. TransLNP adopts a pre-training followed by fine-tuning training approach shown in Fig. 2A. Despite the scarcity of labeled data with LNPs properties in the LNPs field, we can leverage various molecular structure information from other small molecules to train the model. The pre-training task utilizes masked language modeling: randomly masking 15$\%$ of the atoms and introducing mask tokens. Subsequently, an embedding layer is used to map the atomic sequences to representations of atomic types, providing each atom with semantic information. Equation 1 illustrates the process of self-attention mechanism addressing interactions between atoms:


(1) \begin{align*}& attention = \text{softmax}\left(\frac{QK}{\sqrt{d}} + bias\right)V.\end{align*}

The masked atomic types are linearly represented as Queries (Q), Keys (K), and Values (V) in the self-attention module. $\sqrt{d}$ represents the dimensions of vectors Q, K, and V. The attention bias in the self-attention mechanism is first derived by extracting the atomic distance matrix and atomic bond type 3D matrix from the molecular SMILES, with the third dimension expanded into feature dimensions. These matrices are then initialized with a Gaussian distribution. Finally, a trainable gate variable is introduced to fuse the two matrices, resulting in the final bias.

After considering the relationships between atoms, uniform noise is introduced and added to the 3D coordinates of the molecule. This process of coordinate updating effectively incorporates the noise into the molecular representation. In the pretraining phase, atomic type heads, atomic coordinate heads, and atomic pairwise Euclidean distance heads are employed to predict masked atomic types, coordinates, and relative distances, respectively. Smooth L1 is used as the loss function for predicting Euclidean distances and masked atomic coordinates, while cross-entropy is used for predicting atomic types. During fine-tuning, the pretrained model is loaded, following the data processing procedures from pretraining. Finally, molecular prediction heads are used for transfection efficiency prediction.

#### Molecule Mamba

Mamba is a linear-time sequence modeling method based on selective state spaces [35]. Variants of Mamba have demonstrated outstanding performance in natural language processing, image processing, remote sensing, and audio domains [36–39]. State Space Sequence Models (SSMs) [40] form the theoretical foundation of Mamba, capable of mapping a one-dimensional function or sequence $u(t)$ to $y(t) \in \mathbb{R}$ through a hidden state $x(t)$. This mapping can be represented by the following linear ordinary differential equation shown in equation 2:


(2) \begin{align*}& \dot{x}(t) = Ax(t) + Bu(t), \quad y(t) = Cx(t),\end{align*}

where the state matrix $A \in \mathbb{R^{N \times N}}$ serves as the evolution parameter and $B \in \mathbb{R^{N \times 1}}$, $C \in \mathbb{R^{1 \times N}}$ act as projection parameters, representing the implicit latent state.

Due to its combination of recurrent neural networks and CNNs, Mamba has advantages in handling long sequence data. The SMILES representation of ionizable lipids can be viewed as a long sequence for processing. Therefore, we first apply Mamba to the field of molecular property prediction, using one-dimensional SMILES representation of ionizable lipids as sequence inputs. Figure 2B illustrates the overall process of Molecule Mamba. Molecule Mamba involves four parameters $(Delta(\Delta ), A, B, C)$. Firstly, the feature dimension of the SMILES representation molecule sequence is doubled through linear projection. After the split operation, the SMILES representation molecule is represented in two parts. One part undergoes one-dimensional convolution and Sliu activation function to extract molecular features. The use of convolution is to compensate for the SSM’s limitation in modeling local patterns and to provide a content-aware foundation for parameter generation in the SSM. After linear projection and split operation, parameters (Delta, B, C) are obtained. Delta is updated through softplus. At the same time, parameters (A, D) are initialized, where A and D are independent of the input. $A$ is discretized using zero-order hold discretization, while $B$ is discretized using a simplified Euler discretization shown in Equation 3.


(3) \begin{align*}& \overline{A} = \exp(\Delta A), \overline{B} = x + \Delta \cdot f(x, u) .\end{align*}

Combining equation 2 with the scanning operation, the sequential processing of elements in the SMILES representation molecule involves updating atoms at each time step based on the cumulative effect of the previous atom and the current input, effectively propagating information across the entire sequence. Equation 4 describes this process. Finally, the output is the multiplication of another portion of the input with the output of the scanning operation.


(4) \begin{align*}& \dot{x}(t) = \overline{A}x(t) + \overline{B}u(t), \quad y(t) = Cx(t),\end{align*}

#### Mol-attention mechanism block

The purpose of the mol-attention mechanism block is to integrate the output features of the molecule 3D Transformer and molecule Mamba, and introduce an attention mechanism to explain the model’s attention to each atom. The output feature $ z_{1} $ of the molecule 3D Transformer has a size of $ [n,512] $, where $ n $ represents the number of atoms in the ionizable lipid. Meanwhile, the output feature $ z_{2} $ of molecule Mamba has a size of $ [m,512] $, where $ m $ denotes the sequence length of the SMILES representation molecule. Apart from containing atomic features, $ z_{2} $ also includes features of characters in the SMILES representation molecule. Before concatenating the features, all atomic features $ z_{2}^{\prime} $ need to be extracted from $ z_{2} $ to match the size of $ z_{1} $. Therefore each atomic feature in $ z_{2}^{\prime} $ corresponds to one atomic feature in $ z_{1} $, ensuring correct alignment during feature fusion. Like attention mechanisms in the image domain [41–43], the mol-attention mechanism block aims to weight each atom, compressing the fused features containing global information of size $ n \times 1024 $ directly into a $ n \times 1 $ feature vector $ z $. The first fully connected layer $W_{1}$ compresses the 1024 concatenated features into $ 1024/r $ channels to reduce computational complexity, where $ r $ denotes the compression ratio. Following a ReLU non-linear activation layer $\delta $, the second fully connected layer $W_{2}$ produces weights $attention\ scores $ through a Sigmoid activation $\sigma $. The dimension of $attention\ scores$ obtained is $ n \times 1 $, which is shown as Equation 5:


(5) \begin{align*}& scores =\sigma(W_{2} \delta (W_{1}\text{concat}(z_{1}, z_{2}^{\prime}))).\end{align*}

Finally, the scale operation and a regression layer are applied to predict the transfection efficiency, which is shown as Equation 6:


(6) \begin{align*}& pred =\tanh (W_{2}(\tanh( W_{1}(z \cdot scores)))),\end{align*}

where $\tanh $ denotes the tanh activation function.

### Loss function

The loss function of the TransMA model combines the MSE loss function and the triplet loss function, constructing a hybrid loss function. Minimizing Triplet loss brings the features of similar molecules closer and pushes apart those with different predicted values. Combining triplet loss with MSE loss strengthens molecular similarity learning and improves prediction accuracy. Equation 7 is shown as


(7) \begin{align*}& hybrid{\_}loss = \frac{1}{N} \sum_{i=1}^{N} (y_{i} - \hat{y}_{i})^{2} + \beta \times \mathcal{L}_{\text{triplet}}(z_{1}, z_{2}^{\prime}),\end{align*}

where $\beta $ is a weight parameter, $N$ is the number of samples, $y_{i}$ and $\hat{y}_{i}$, respectively, represent the true values and the predicted values, and the definitions of $z_{1}$ and $z_{2}^{\prime}$ are consistent with equation 5. Equation 8 describes the triplet loss:


(8) \begin{align*}& \begin{split} \mathcal{L}_{\text{triplet}} = \frac{1}{\text{np} + \epsilon} \sum_{i=1}^{2b} \sum_{j=1}^{2b} \sum_{k=1}^{2b} L\cdot \text{mask}_{ijk},\\ L=\max(d(z_{1i}, z_{1j}) - d(z_{1i}, z_{2k}) + \text{margin}, 0), \end{split}\end{align*}

where $b$ represents the batch size, $z_{1i}$ represents the feature extracted from the molecule 3D Transformer for the $i$th sample, $z_{2k}^{\prime}$ denotes the feature extracted from the molecule Mamba for the $k$th sample, $d(\cdot )$ denotes the Euclidean distance, $\text{np}$ is the number of positive losses, $\epsilon $ is a small number used for numerical stability, $margin$ is a hyperparameter controlling the margin size for effective triplets, and $\text{mask}_{ijk}$ is a triplet mask used to filter out invalid triplets.

The Triplet Loss first computes the Euclidean distance between $z_{1}$ and $z_{2}$. This loss value is calculated as the difference between the distance from the anchor sample to the positive sample and the distance from the anchor sample to the negative sample, with a margin added, and then taking the maximum value. Subsequently, invalid triplets are filtered out using a triplet mask, and the average loss value is computed as the final result of the triplet Loss. Triplet loss maps the molecular 3D structural features extracted by the molecule 3D Transformer and molecule Mamba, along with the one-dimensional sequence features, to a unified embedding space.



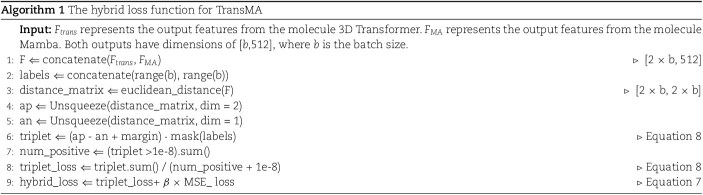



## Experimental section

### Dataset

The dataset for TransMA predicting LNPs transfection efficiency comprises two main components. One part is utilized for pretraining the molecule 3D Transformer, while the other part is used for training the molecule Mamba and fine-tuning the molecule 3D Transformer. The dataset used for pretraining the molecule 3D Transformer is sourced from Unimol [44], which comprises 19 datasets (The 19 datasets include Targetmol (link), Chemdiv (link), Enamine (link), Chembridge (link), Life Chemical (link), Specs (link), Vitas-M (link), InterBioScreen (link), Maybridge (link), Bionet-Key Organics (link), Asinex (link), UkrOrgSynthesis (link), Eximed (link), HTS Biochemie Innovationen (link), Princeton BioMolecular (link), Otava (link), Alinda Chemical (link), Analyticon (link), ZINC (link), and ChemBL (link)). The dataset utilized for training the molecule Mamba and fine-tuning the molecule 3D Transformer is obtained from AGILE (https://github.com/bowang-lab/AGILE), consisting of 1200 ionizable lipid SMILES representation molecules along with their corresponding transfection efficiency data in Hela and RAW 264.7 cell lines. Hela cells are primarily used to assess high transfection efficiency in nonimmune cells, making them suitable for gene therapy and vaccine development, while the RAW cell line simulates transfection in immune cells, providing valuable data on the role and performance of LNPs in immune responses. The reason for choosing Hela and RAW cell lines lies in their significant differences in LNPs-mRNA transfection efficiency, with immune cells being more difficult to transfect than Hela cells. By obtaining data from these two cell lines, TransMA can learn the characteristics of different transfection efficiency ranges, enhancing the generalization ability and prediction accuracy of the TransMA model,

Ionizable lipids comprises 20 head groups, 12 carbon chains with ester bonds, and five carbon chains with isocyanide head groups. High-throughput synthesis of LNPs entails mixing an aqueous phase containing mRNA and an ethanol phase containing lipids using the OT-2 pipetting robot. The aqueous phase, prepared in pH 4.0 10 mM sodium citrate buffer, contains firefly luciferase mRNA, Cre recombinase mRNA, or EGFP-mRNA. Meanwhile, the ethanol phase comprises a mixture of 1200 ionizable lipids and fixed proportions of helper phospholipids (DOTAP, DOPE, cholesterol, and C14-PEG 2000), with a lipid-to-mRNA weight ratio of 10:1. Transfection efficiency data were obtained by applying these 1200 mRNA-LNPs to Hela and RAW 264.7 cell lines for in vitro transfection experiments.

### Experimental processing

The tasks of molecule 3D Transformer pretraining include using random positions as corrupted input 3D positions and training the model to predict the correct positions, employing different heads to predict distances between corrupted atom pairs, the correct coordinates of corrupted atoms, and to mask and predict the atom types of corrupted atoms. Atomic type prediction employs the cross-entropy loss function with a weight of 1. Prediction related to atomic coordinates and interatomic distances utilizes the smooth L1 loss function with weights set to 5 and 10, respectively. Pretraining parameters are set as follows: model layers are 15, batch size is 128, atom types are 30, learning rate is adjusted using linear decay, and the optimizer is Adam with $\epsilon $. Parameters for molecule 3D Transformer fine-tuning are as follows: model layers are 16, batch size is 4, epochs are set to 200, and the initial learning rate is set to 1e-5.

Molecule Mamba first tokenizes 1200 ionizable lipid SMILES molecular representation. The tokenizer used is ChemBERTa-77M-MTR from Deepchem. The purpose of tokenization is to segment SMILES molecule repren into tokens and convert these tokens into numerical representations that the model can process. The parameters of the Molecule Mamba model are set as follows: the feature dimension is 512, the number of layers is 2, the vocabulary size is 100, and the training process parameters are the same as molecule 3D Transformer fine-tuning. In TransMA’s hybrid loss function, the parameter $\beta $ is set to 6 during the scaffpld data splitting and 3 during the cliff data splitting.

### Comparison with representative deep learning-based molecular property prediction models

To demonstrate the superiority of the proposed method, compare the predictive transfection efficiency accuracy of TransMA with advanced molecular property prediction models based on graph CNNs and Transformer on the LNPs dataset. The compared five models include AGILE, large-scale self-supervised pretraining for molecular property prediction model named ChemBERTa [26], self-supervised graph neural network framework named MolCLR [45], and geometry enhanced molecular representation learning model named GEM [46], TransLNP.

**Figure 3:**
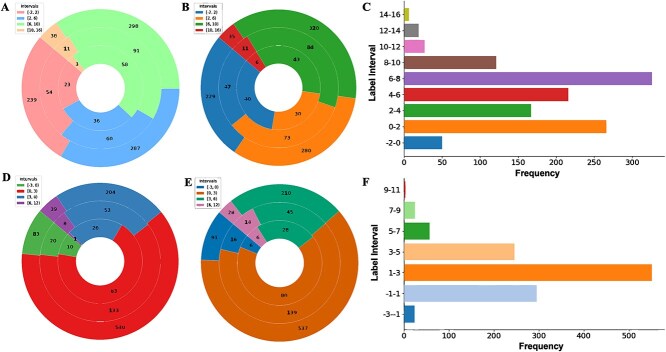
The distribution of the transfection efficiency dataset across two data splitting methods in Hela and RAW 264.7 cell lines is shown, with pie charts representing the training (outer ring), validation (middle ring), and test (inner ring) sets, where subfigures A-C show Hela cell splits (scaffold-based, cliff-based, and overall), and subfigures D-F show the corresponding splits for RAW 264.7 cells.

The LNPs dataset includes 1200 ionizable lipid SMILES molecules and transfection efficiency data obtained from Hela and RAW 264.7 cell lines. In model comparison, the LNPs dataset is divided into cliff [47] and scaffold data splitting approaches. Figure 3 represents LNPs dataset distributions under two data splitting methods in Hela and RAW 264.7 cell lines. Scaffold splitting divides LNPs dataset into training set, validation set and testing set as shown in Fig. 3A and Fig. 3D. Cliff splitting utilizes molecular extended connectivity fingerprints (ECFP) descriptors to represent their structures, employing algorithms like spectral clustering to partition molecules into five clusters. Cliff splitting divides LNPs dataset into training set, validation set, and testing set as shown in Fig. 3B and Fig. 3E. For each cluster, a stratified sampling strategy is employed to allocate 10$\%$ of the molecules to the testing set and other molecules to the training set and validation set in an 8:2 ratio. Cliff splitting ensures that training and test sets contain the proportion of cliff molecule pairs in the training and testing sets while preserving information about molecular structural similarity. Scaffold splitting involves grouping molecules based on their core structural scaffolds, ensuring that both training and test sets contain molecules from diverse scaffold structures [48]. Cliff splitting considers the similarity of data, while scaffold splitting considers the diversity of data. Therefore, combining both cliff and scaffold data splitting methods can comprehensively validate the model’s generalization capability.

**Figure 4:**
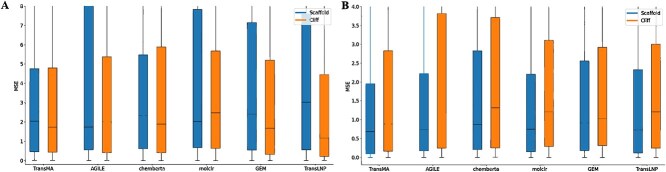
The comparison of TransMA's performance in predicting transfection efficiency against five different models under scaffold-based and cliff-based data splitting methods for Hela and RAW 264.7 cell lines is presented, with subfigure A showing box plots for the Hela cell line and subfigure B showing similar comparisons for the RAW 264.7 cell line.

**Figure 5:**
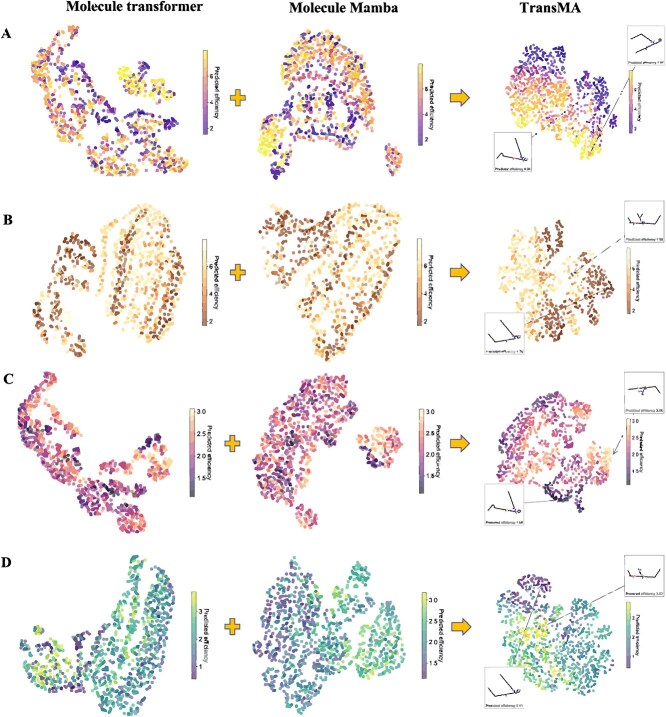
The UMAP visualization of TransMA's multimodal feature fusion process, based on the Molecule 3D Transformer and Molecule Mamba under scaffoldbased and cliff-based data splitting methods, is shown for Hela and RAW 264.7 cell lines, with subfigures A and B representing fusion features in Hela cells (scaffold-based and cliff-based, respectively), and subfigures C and D showing the corresponding splits in RAW 264.7 cells.

Table 1 presents the results of TransMA and the compared models in predicting transfection efficiency on the LNPs dataset. Evaluation metrics for assessing prediction performance include MSE, mean absolute error (MAE), the coefficient of determination R$^{2}$, and the Pearson correlation coefficient (PCC). The results demonstrate that TransMA exhibits superior performance compared with the five other models. Figure 4 shows the boxplots of MSE for six models in Hela and RAW 264.7 cell lines. It can be observed that TransMA exhibits the lowest MSE in predicting transfection efficiency on both cell lines, indicating the smallest fluctuation in prediction errors. Figure 5 show TransMA’s capability to extract multimodal fused molecular features under the scaffold and cliff data splitting methods in Hela and RAW 264.7 cell lines. It can be observed that the molecular features extracted by molecule 3D Transformer and molecule Mamba exhibit discrete states in different transfection efficiency intervals. After feature fusion by TransMA, the molecular features of TransMA show localization, meaning that the distribution of features for the same transfection efficiency interval is close. Figure 5 indicates that TransMA has successfully established a mapping relationship between the ionizable lipid structure and transfection efficiency.

**Table 1:** The results of predicting LNPs transfection efficiency in both Hela and Raw 264.7 cell lines by six models under scaffold and cliff data splitting methods are presented. Prediction accuracy is assessed using MSE (smaller values indicate better performance), MAE(smaller values indicate better performance), R$^{2}$ (larger values indicate better performance), and PCC (larger values indicate better performance). “BERTa” refers to the chemBERTa model, while “RAW” refers to Raw 264.7 cells. The best results are highlighted in bold

Splitting		TransMA		AGILE		BERTa		MolCLR		GEM		TransLNP	
		Hela	RAW	Hela	RAW	Hela	RAW	Hela	RAW	Hela	RAW	Hela	RAW
Scaffold	MSE$\downarrow $	**3.64**	**1.63**	6.38	2.07	4.38	2.11	6.18	2.00	5.89	2.24	5.58	1.88
	MAE$\downarrow $	**1.57**	**0.98**	1.92	1.11	1.70	1.13	1.98	1.09	1.92	1.16	1.92	1.06
	$R^{2}$ $\uparrow $	**0.49**	**0.23**	0.11	0.02	0.39	0.01	0.12	0.05	0.18	-0.05	0.22	0.12
	PCC $\uparrow $	**0.75**	**0.54**	0.46	0.24	0.63	0.36	0.42	0.25	0.48	0.24	0.47	0.37
Cliff	MSE $\downarrow $	**4.36**	**2.88**	5.94	2.90	5.34	2.98	5.96	3.07	5.51	3.17	4.47	3.06
	MAE $\downarrow $	**1.62**	**1.24**	1.79	1.33	1.78	1.34	1.89	1.35	1.72	1.33	1.55	1.30
	$R^{2}$ $\uparrow $	**0.61**	**0.09**	0.47	0.08	0.53	0.06	0.47	0.05	0.51	0.00	0.60	0.04
	PCC $\uparrow $	**0.79**	**0.34**	0.69	0.30	0.73	0.27	0.69	0.29	0.74	0.29	0.78	0.27

### Ablation experiment

TransMA’s ability to extract molecular features comes from both molecule 3D Transformer and molecule Mamba. To demonstrate the superiority of multimodal feature extraction for molecules, a ablation experiment is designed: comparing the accuracy of predicting transfection efficiency between TransMA, molecule 3D Transformer, and molecule Mamba under scaffold and cliff data splitting methods in Hela and RAW 264.7 cell lines. In the ablation experiment, TransMA, molecule 3D Transformer, and molecule Mamba ensure consistency in training set and testing set, as well as parameter settings. Table 2 presents the performance of TransMA, molecule 3D Transformer, and molecule Mamba in predicting transfection efficiency under two different data splitting methods in Hela and RAW 264.7 cell lines. The results demonstrate that TransMA outperforms both molecule 3D Transformer and molecule Mamba in predicting transfection efficiency. The ablation experiment indicates that the fusion of molecule 3D Transformer and molecule Mamba for extracting multidimensional molecular structural features is a key factor in achieving superior performance in the task of transfection efficiency prediction.

**Table 2:** The results of predicting LNPs transfection efficiency by TransMA, molecule 3D Transformer, and molecule Mamba under scaffold and cliff data splitting methods in Hela and RAW 264.7 cell lines are presented. The best results are highlighted in bold

Splitting	Scaffold						Cliff					
Method	TransMA		Molecule Transformer		Molecule Mamba		TransMA		Molecule Transformer		Molecule Mamba	
	Hela	RAW	Hela	RAW	Hela	RAW	Hela	RAW	Hela	RAW	Hela	RAW
MSE $\downarrow $	**3.64**	**1.63**	5.13	1.77	6.38	1.90	**4.36**	**2.88**	4.63	3.02	5.29	3.00
MAE $\downarrow $	**1.57**	**0.98**	1.88	1.02	2.17	1.07	**1.62**	**1.24**	**1.62**	1.29	1.74	1.30
$R^{2}$ $\uparrow $	0.49	**0.23**	**0.56**	0.17	0.11	0.10	**0.61**	**0.09**	0.59	0.05	0.53	0.06
PCC $\uparrow $	**0.75**	**0.54**	0.28	0.43	0.39	0.34	**0.79**	**0.34**	0.78	0.27	0.74	0.30

### Analysis of model interpretability

In this section, the interpretability of the model is analyzed to verify whether TransMA can identify the key atoms in ionizable lipids that affect transfection efficiency. First, identifying the locations of key atoms in ionizable lipids is fundamental for interpretability analysis. Therefore, constructing transfection cliffs is employed to pinpoint key atoms. Concurrently, the mol-attention mechanism block calculates the influence scores of all atoms in ionizable lipids on transfection efficiency. If the high-scoring atoms identified by the mol-attention mechanism align with the key atoms found through the construction of transfection cliffs, it proves that the model can accurately recognize the key atoms in ionizable lipids that impact transfection efficiency.

#### Transfection cliffs

Transfection cliff molecular pairs refer to ionizable lipid molecules that are structurally very similar but have significantly different transfection efficiencies. The phenomenon of transfection cliffs exists in LNP datasets and can be observed through UMAP plots where points that are close in space exhibit large differences in mapped colors. Despite the structural similarity of transfection cliff molecular pairs, there are differences in their atomic compositions, which lead to significant differences in transfection efficiency. Therefore, the atoms that differ between the structures of transfection cliff molecular pairs are considered key atoms affecting transfection efficiency. To quantify the similarity of ionizable lipid molecules, similarity scores based on substructure similarity, scaffold similarity, and SMILES string similarity are constructed [49]. Substructure similarity and scaffold similarity are determined by calculating the Tanimoto coefficient of the ECFP [50, 51] and the Molecular ACCess System (MACCS) keys [52, 53] for the molecular graph frameworks, respectively. SMILES string similarity is assessed by calculating the Levenshtein distance [54] between the SMILES string representations of the molecules. Equation 9 calculates the Tanimoto coefficient shown as


(9) \begin{align*}& \text{Tanimoto coefficient} = \frac{c}{a + b - c}.\end{align*}

For substructure similarity, $a$ counts the number of set bits (1s) in molecule A’s ECFP fingerprint, $b$ does the same for molecule B, and $c$ counts the number of common set bits in both fingerprints. For scaffold similarity, $a$ counts set bits in molecule A’s MACCS keys, $b$ does the same for molecule B, and $c$ counts common set bits in both molecules’ MACCS keys. Equation 10 computes SMILES molecular similarity shown as


(10) \begin{align*}& \text{smis} = 1 - \frac{d(s_{1}, s_{2})}{\max(|s_{1}|, |s_{2}|)} ,\end{align*}

where $d(s1, s2)$ is the Levenshtein distance between the SMILES strings of two molecules.

Similar molecule pairs are defined as pairs with a structural similarity greater than 0.9. Equation 11 describes the structural similarity, which is the average of substructure similarity, scaffold similarity, and SMILES string similarity shown as


(11) \begin{align*}& \text{structure similarity} = \frac{subs + scas + smis}{3} ,\end{align*}

where $subs$ represents substructure similarity, $scas$ represents scaffold similarity, and $smis$ represents SMILES string similarity. Since the transfection efficiency in the LNPs dataset has been log2-transformed, similar molecule pairs are identified as transfection cliff pairs when the transfection difference exceeds 1. Equation 12 describes the transfection difference (TD) shown as


**Figure 6:**
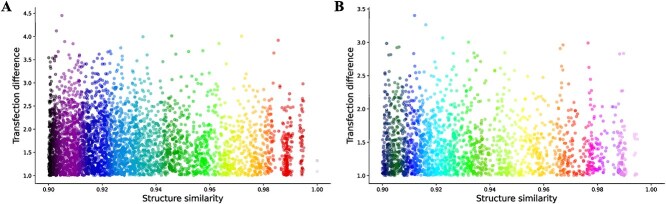
Scatter plots illustrating the relationship between structural similarity and transfection difference for transfection cliff pairs in Hela and RAW 264.7 cell lines are presented, with subfigure A showing the plot for Hela cells and subfigure B showing the plot for RAW 264.7 cells.

(12) \begin{align*}& TD = |\log_{10}(2^{{m_{2}-m1}})|,\end{align*}

where $m_{1}$ and $m_{2}$ represent the transfection efficiencies of the two molecules in the similar pair. A total of 4267 and 2104 transfection cliff pairs are identified in the Hela and RAW 264.7 cell lines, respectively. Figure 6 shows the scatter plots of structural similarity and transfection difference for transfection cliff pairs in the Hela and RAW 264.7 cell lines. From the figure, it can be seen that when the structural similarity of the molecules is greater than 0.9, the transfection difference is significant, reaching up to a thousand-fold or even 10 000-fold difference.

#### Model interpretability

**Figure 7:**
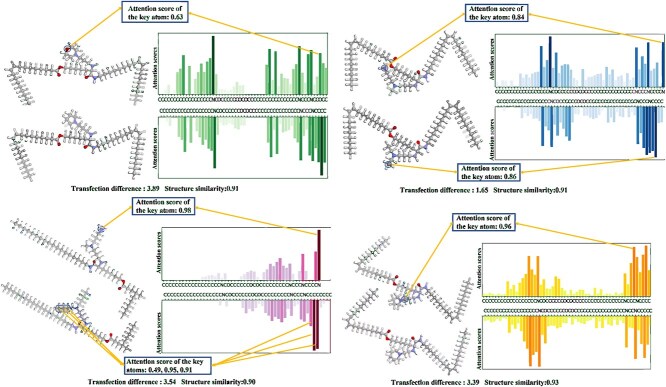
A scatter plot illustrating the distribution of attention scores (ranging from 0 to 1) and highlighting key atoms for transfection cliff pairs, where the transfection difference (expressed as a power of 10) indicates the multiplicative change in transfection efficiency between two molecules, and structural similarity ranges from 0 to 1.

The transfection cliff phenomenon can help identify key atoms in ionizable lipids that affect transfection efficiency. Figure 6 shows that even when the molecular similarity of transfection cliff pairs is close to 1, the transfection difference remains significant. This implies that the one or two differing atoms between the two molecules in the transfection cliff pairs are the cause of the large transfection difference. Therefore, the key atoms are those differing atoms in the transfection cliff pairs. When predicting the transfection efficiency of transfection cliff pairs, the mol-attention mechanism block in TransMA calculates the attention scores for all atoms. Figure 7 shows the attention scores calculated by the mol-attention mechanism block and the key atoms of the transfection cliff pairs. It can be seen that the atoms with the highest attention scores are precisely the key atoms responsible for the differences in the transfection cliff pairs. For example, in Fig. 7, the similarity of the transfection cliff pair is 0.91, and the transfection difference is 1.69. This indicates that the transfection efficiency of one molecule is $10^{1.69}$ times higher than the other. The two molecules differ by only one carbon atom (C) and one nitrogen atom (N) in structure. The attention scores corresponding to the differing C and N atoms are 0.84 and 0.86, significantly higher than the attention scores for other atoms. Therefore, TransMA can identify the key atoms affecting transfection efficiency, demonstrating the interpretability of the model.

### External test

**Figure 8:**
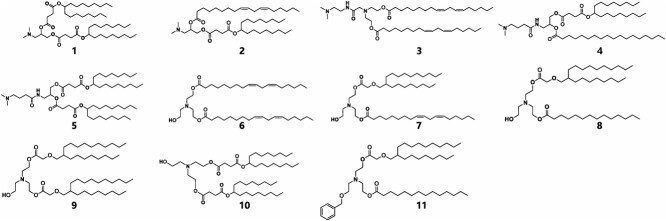
The molecular structures of the 11 ionizable lipids derived from external data sources are presented.

While TransMA achieves accurate predictions of transfection efficiency on the LNPs dataset, we aim to evaluate its generalization capability by testing it on an external dataset without additional training. We compile an external dataset based on published studies, consisting of 15 LNPs with ionizable lipids as the only variable and their transfection efficiency levels. Among these, 11 ionizable lipids, which are from different combinations of six head groups, one ester linkage, and five hydrophobic tails shown in Fig. 8, and their transfection efficiencies [55] are obtained from Balb/c mice using a fixed LNP formulation. The remaining four ionizable lipids and their transfection efficiencies [56–58] are derived from different cell lines. Since the TransMA model is also trained on different cell lines, it possesses the capability to predict transfection efficiency across various cell lines.

**Figure 9:**
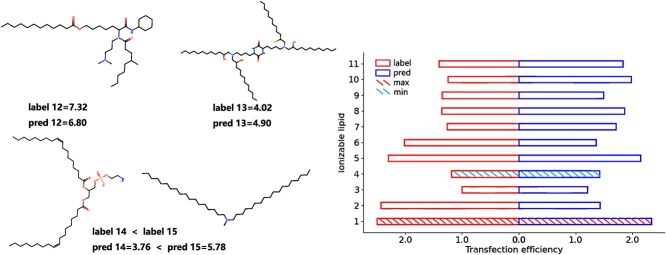
Predicted transfection efficiency results for the first 11 ionizable lipids are shown, along with both molecular structures and predicted results for ionizable lipids 12–15.

Figure 9 shows the results of TransMA directly testing on an external dataset without training. The predicted maximum and minimum transfection efficiencies of lipids 1-11 by TransMA are consistent with the true values, and the order of predicted values is similar to that of true values. For lipids 12 and 13, the prediction error is <1. As for lipids 14 and 15, the authors only provided the transfection efficiency of lipid 14 being lower than that of lipid 15 during collection, which is consistent with the prediction result. TransMA achieves good predictions of transfection efficiency even without training on the external dataset, demonstrating its strong generalization capability.

### Model complexity

The parameter count of TransMA comes from the six-layer Molecular 3D Transformer, the two-layer Molecular Mamba, and the Mol-attention mechanism block shown in Table 3. Table 3 indicates that the majority of TransMA’s parameters come from the molecule 3D Transformer, accounting for 91$\%$ of the total. To demonstrate that TransMA’s superior performance is not merely due to a large number of parameters, Table 2 compares TransMA with the molecule 3D Transformer, showing that TransMA outperforms it despite having a similar number of parameters.

**Table 3:** Parameter counts for different components of TransMA

	Params
Molecule 3D Transformer	47 593 795
Molecule Mamba	3379 201
Mol-attention mechanism block	1050 626

Additionally, increasing the number of layers in Molecule Mamba to 33 results in a parameter count that surpasses that of TransMA. The performance of the 33-layer molecule Mamba on the LNPs dataset is shown in Table 4. The results in Table 4 show that, despite molecule Mamba having more parameters than TransMA, its predictive performance is still inferior to that of TransMA. Finally, an ablation study concerning the mol-attention mechanism block is added. The LNPs property predictions made by TransMA without the mol-attention mechanism block are also shown in Table 4. The ablation study demonstrates that the mol-attention mechanism block not only enables the calculation of atomic scores but also enhances prediction accuracy. In summary, the outstanding performance of TransMA is not due to a simple accumulation of parameters from the molecule 3D Transformer or molecule Mamba.

**Table 4:** Prediction results of the 33-layer molecule Mamba (Params: 54 915 585) and TransMA without the Mol-attention mechanism block under scaffold and cliff data splitting methods in Hela and RAW 264.7 cells on the LNPs dataset

Method	$\text{Mamba}_{\text{33 layers}}$				$\text{TransMA}_{\text{without att}}$			
Splitting	Scaffold		Cliff		Scaffold		Cliff	
Cells	Hela	RAW	Hela	RAW	Hela	RAW	Hela	RAW
MSE $\downarrow $	5.37	1.87	5.08	2.89	4.18	1.98	4.96	3.20
MAE $\downarrow $	1.89	1.06	1.70	1.30	1.70	1.12	1.76	1.29
$R^{2}$ $\uparrow $	0.25	0.12	0.55	0.09	0.42	0.07	0.56	0.00
PCC $\uparrow $	0.50	0.36	0.75	0.34	0.67	0.38	0.77	0.20

## Discussion and conclusion

In this work, we propose an explainable high-accuracy LNPs transfection efficiency prediction model called TransMA. To achieve high-accuracy predictions, TransMA employs a multimodal molecular structure fusion architecture, where the substructure molecule 3D Transformer extracts three-dimensional spatial features of the molecule, and molecule mamba extracts one-dimensional molecular features. To achieve model interpretability and feature fusion, we design the mol-attention mechanism block. This block can integrate multidimensional features and reveal atom-level structure–transfection relationships based on the molecular channel attention mechanism. Compared with advanced molecular graph convolutional networks and Transformer models, TransMA achieves the highest accuracy in predicting transfection efficiency under the scaffold and cliff data splitting methods in Hela and RAW 264.7 cell lines. Moreover, we introduce transfection cliff pairs. The results demonstrate that the atoms with high attention scores computed by the mol-attention mechanism block correspond to the key atoms in the transfection cliff pairs, showing that TransMA can identify the key atoms affecting transfection efficiency. Additionally, the external test results indicate that even on the untrained external dataset, TransMA’s predicted values maintain a consistent order with the actual transfection efficiencies.

Although TransMA has demonstrated excellent performance in predicting transfection efficiency, there are still limitations in prediction accuracy due to the scarcity of LNPs datasets. Additionally, since ionizable lipids are composed mainly of head and tail groups, different ionizable lipids tend to have high structural similarity, making the occurrence of transfection cliffs more likely. These transfection cliffs increase the difficulty for TransMA to capture the mapping relationship between molecular structure and transfection efficiency. Therefore, future work should focus on addressing the transfection cliff phenomenon to further improve prediction accuracy. Although TransMA has validated the accuracy of predicting transfection efficiency in cultured cells, translating this to therapeutic settings remains challenging due to differences between *in vitro* and *in vivo* environments. Factors such as LNP stability, immune responses, cell type diversity, and tissue distribution can affect transfection efficiency *in vivo*, requiring greater generalization from the model. However, TransMA has shown strong generalization ability in external data testing. We believe that with further data and optimizations, TransMA can play a crucial role in improving LNP delivery efficiency in clinical settings.

**Key Points**

- An explainable LNPs transfection efficiency prediction model called TransMA.
- TransMA employs a multimodal molecular structure fusion architecture.
- TransMA reveals the atomic-level structure–transfection relationships.
- TransMA achieves state-of-the-art performance on the largest current LNPs dataset.

## Data Availability

The code, model, and data are made publicly available at https://github.com/wklix/TransMA/tree/master.
